# 3-(3-Fluoro­phenyl­sulfin­yl)-5-iodo-2,7-dimethyl-1-benzofuran

**DOI:** 10.1107/S1600536812037944

**Published:** 2012-09-08

**Authors:** Hong Dae Choi, Pil Ja Seo, Uk Lee

**Affiliations:** a Department of Chemistry, Dongeui University, San 24 Kaya-dong, Busanjin-gu, Busan 614-714, Republic of Korea; b Department of Chemistry, Pukyong National University, 599-1 Daeyeon 3-dong, Nam-gu, Busan 608-737, Republic of Korea

## Abstract

In the title compound, C_16_H_12_FIO_2_S, the 3-fluoro­phenyl ring makes a dihedral angle of 76.47 (6)° with the mean plane [r.m.s. deviation = 0.013 (2) Å] of the benzofuran fragment. In the crystal, mol­ecules are linked by weak C—H⋯O hydrogen bonds,forming chains along the *b*-axis direction, and an I⋯O contact [3.204 (2) Å]. The crystal structure also exhibits slipped π–π inter­actions between the 3-fluoro­phenyl rings of neighbouring mol­ecules [centroid–centroid distance = 3.683 (3) Å and slippage = 1.708 (3) Å].

## Related literature

For background information and the crystal structures of related compounds, see: Choi *et al.* (2008 ▶, 2011 ▶). For a review of halogen bonding, see: Politzer *et al.* (2007 ▶). 
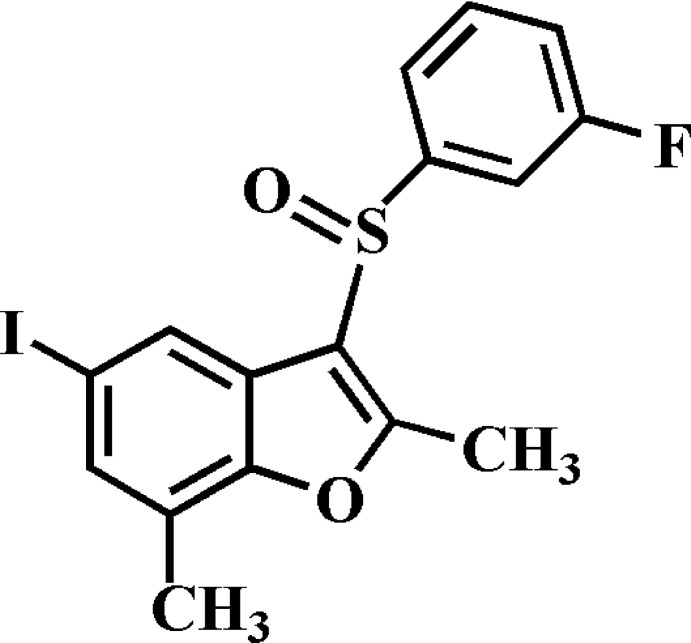


## Experimental

### 

#### Crystal data

C_16_H_12_FIO_2_S
*M* *_r_* = 414.22
Monoclinic, 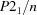

*a* = 8.3285 (3) Å
*b* = 15.1807 (5) Å
*c* = 12.2471 (4) Å
β = 90.250 (2)°
*V* = 1548.42 (9) Å^3^
*Z* = 4
Mo *K*α radiation
μ = 2.21 mm^−1^
*T* = 173 K
0.43 × 0.18 × 0.14 mm

#### Data collection

Bruker SMART APEXII CCD diffractometer
Absorption correction: multi-scan (*SADABS*; Bruker, 2009 ▶) *T*_min_ = 0.452, *T*_max_ = 0.740
14445 measured reflections
3815 independent reflections
3409 reflections with *I* > 2σ(*I*)
*R*_int_ = 0.035

#### Refinement

*R*[*F* ^2^ > 2σ(*F* ^2^)] = 0.026
*wR*(*F* ^2^) = 0.065
*S* = 1.05
3815 reflections
192 parameters
H-atom parameters constrained
Δρ_max_ = 0.48 e Å^−3^
Δρ_min_ = −0.71 e Å^−3^

### 

Data collection: *APEX2* (Bruker, 2009 ▶); cell refinement: *SAINT* (Bruker, 2009 ▶); data reduction: *SAINT*; program(s) used to solve structure: *SHELXS97* (Sheldrick, 2008 ▶); program(s) used to refine structure: *SHELXL97* (Sheldrick, 2008 ▶); molecular graphics: *ORTEP-3* (Farrugia, 2012 ▶) and *DIAMOND* (Brandenburg, 1998 ▶); software used to prepare material for publication: *SHELXL97*.

## Supplementary Material

Crystal structure: contains datablock(s) global, I. DOI: 10.1107/S1600536812037944/im2399sup1.cif

Structure factors: contains datablock(s) I. DOI: 10.1107/S1600536812037944/im2399Isup2.hkl

Supplementary material file. DOI: 10.1107/S1600536812037944/im2399Isup3.cml

Additional supplementary materials:  crystallographic information; 3D view; checkCIF report

## Figures and Tables

**Table 1:** Hydrogen-bond geometry (Å, °)

*D*—H⋯*A*	*D*—H	H⋯*A*	*D*⋯*A*	*D*—H⋯*A*
C14—H14⋯O2^i^	0.95	2.43	3.292 (3)	151

Symmetry code: (i) 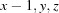.

**Fig. 1.**
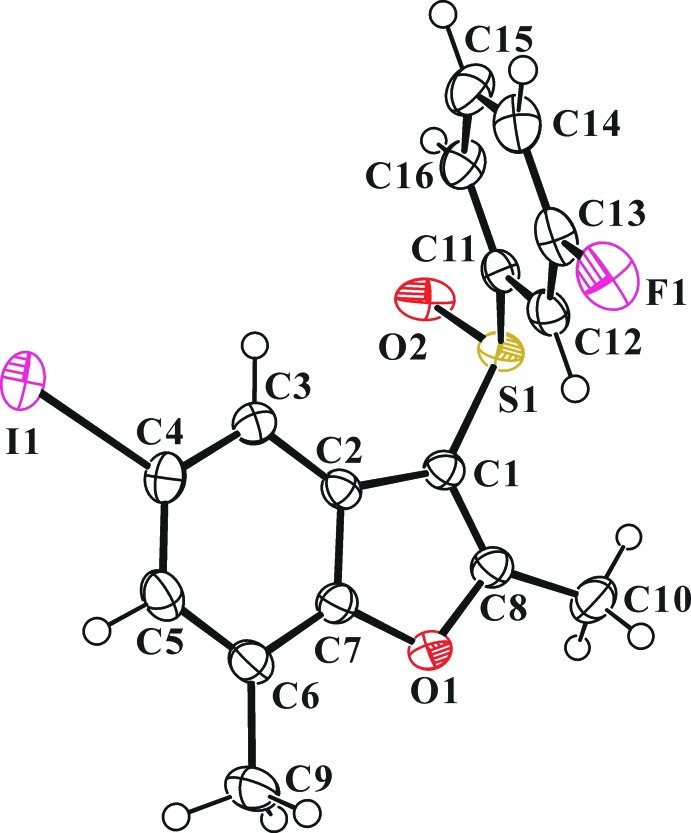
Molecular structure of the title compound with displacement ellipsoids drawn at the 50% probability level. H atoms are presented as small spheres of arbitrary radius.

**Fig. 2.**
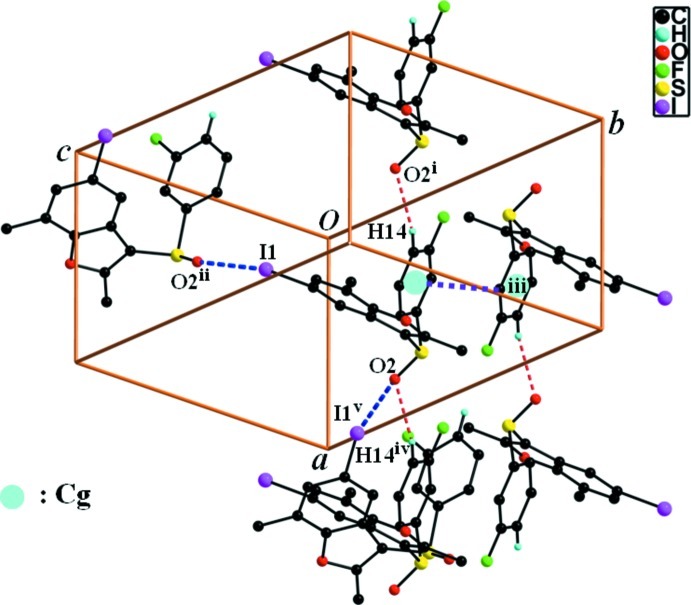
A view of the C—H···O, I···O, and π–π interactions (dotted lines) in the crystal structure of the title compound. H atoms not participating in hydrogen-bonding were omitted for clarity. [Symmetry codes: (i) x - 1, y, z; (ii) x -1/2, - y + 1/2, z + 1/2; (iii) - x + 1, - y + 1, - z; (iv) x + 1, y, z; (v) x + 1/2, - y + 1/2, z - 1/2.]

## supplementary crystallographic information

### Comment

As a part of our ongoing study of 5-iodo-2,7-dimethyl-1-benzofuran derivatives containing 3-phenylsulfinyl (Choi *et al.*, 2008) and 3-(4-fluorophenylsulfinyl) (Choi *et al.*, 2011) substituents, we report herein the crystal structure of the title compound.

In the title molecule (Fig. 1), the benzofuran unit is essentially planar, with a mean deviation of 0.013 (2) Å from the least-squares plane defined by the nine constituent atoms. The dihedral angle between the 3-fluorophenyl ring and the mean plane of the benzofuran fragment is 76.47 (6)°. In the crystal structure (Fig. 2), molecules are connected by weak C—H···O hydrogen bonds (Table 1), and by an I···O halogen-bonding between the iodine and the oxygen of the S═O unit [I1···O2^ii^ = 3.204 (2)Å, C4—I1···O2^ii^ = 165.03 (7)] (Politzer *et al.*, 2007). The crystal packing (Fig. 2) also exhibits slipped π–π interactions between the 3-fluorophenyl rings of neighbouring molecules, with a Cg—Cg^iii^ distance of 3.683 (3) Å and an interplanar distance of 3.263 (3) Å resulting in a slippage of 1.708 (3) Å (Cg is the centroid of the C11–C16 3-fluorophenyl ring).

### Experimental

3-Chloroperoxybenzoic acid (77%, 202 mg, 0.9 mmol) was added in small portions to a stirred solution of 3-(3-fluorophenylsulfanyl)-5-Iodo-2,7-dimethyl-1-benzofuran (318 mg, 0.8 mmol) in dichloromethane (30 mL) at 273 K. After being stirred at room temperature for 4h, the mixture was washed with saturated sodium bicarbonate solution and the organic layer was separated, dried over magnesium sulfate, filtered and concentrated at reduced pressure. The residue was purified by column chromatography (hexane–ethyl acetate, 2:1 v/v) to afford the title compound as a colorless solid [yield 74%, m.p. 445–446 K; *R*_f_ = 0.66 (hexane–ethyl acetate, 2:1 v/v)]. Single crystals suitable for X-ray diffraction were prepared by slow evaporation of a solution of the title compound in ethyl acetate at room temperature.

### Refinement

All H atoms were positioned geometrically and refined using a riding model, with C—H = 0.95 Å for aryl and 0.98 Å for methyl H atoms. *U*_iso_(H) = 1.2*U*_eq_(C) for aryl and 1.5*U*_eq_(C) for methyl H atoms. The positions of methyl hydrogens were optimized rotationally.

### Crystal data

**Table:**

C_16_H_12_FIO_2_S	*F*(000) = 808
*M**_r_* = 414.22	*D*_x_ = 1.777 Mg m^−^^3^
Monoclinic, *P*2_1_/*n*	Mo *K*α radiation, λ = 0.71073 Å
Hall symbol: -P 2yn	Cell parameters from 7687 reflections
*a* = 8.3285 (3) Å	θ = 2.7–28.3°
*b* = 15.1807 (5) Å	µ = 2.21 mm^−^^1^
*c* = 12.2471 (4) Å	*T* = 173 K
β = 90.250 (2)°	Block, colourless
*V* = 1548.42 (9) Å^3^	0.43 × 0.18 × 0.14 mm
*Z* = 4	

### Data collection

**Table:**

Bruker SMART APEXII CCD diffractometer	3815 independent reflections
Radiation source: rotating anode	3409 reflections with *I* > 2σ(*I*)
Graphite multilayer monochromator	*R*_int_ = 0.035
Detector resolution: 10.0 pixels mm^-1^	θ_max_ = 28.3°, θ_min_ = 2.1°
φ and ω scans	*h* = −11→11
Absorption correction: multi-scan (*SADABS*; Bruker, 2009)	*k* = −20→20
*T*_min_ = 0.452, *T*_max_ = 0.740	*l* = −12→16
14445 measured reflections	

### Refinement

**Table:**

Refinement on *F*^2^	Primary atom site location: structure-invariant direct methods
Least-squares matrix: full	Secondary atom site location: difference Fourier map
*R*[*F*^2^ > 2σ(*F*^2^)] = 0.026	Hydrogen site location: difference Fourier map
*wR*(*F*^2^) = 0.065	H-atom parameters constrained
*S* = 1.05	*w* = 1/[σ^2^(*F*_o_^2^) + (0.0282*P*)^2^ + 0.8484*P*] where *P* = (*F*_o_^2^ + 2*F*_c_^2^)/3
3815 reflections	(Δ/σ)_max_ = 0.001
192 parameters	Δρ_max_ = 0.48 e Å^−^^3^
0 restraints	Δρ_min_ = −0.71 e Å^−^^3^

### Special details

**Table:**

Geometry. All esds (except the esd in the dihedral angle between two l.s. planes) are estimated using the full covariance matrix. The cell esds are taken into account individually in the estimation of esds in distances, angles and torsion angles; correlations between esds in cell parameters are only used when they are defined by crystal symmetry. An approximate (isotropic) treatment of cell esds is used for estimating esds involving l.s. planes.
Refinement. Refinement of F^2^ against ALL reflections. The weighted R-factor wR and goodness of fit S are based on F^2^, conventional R-factors R are based on F, with F set to zero for negative F^2^. The threshold expression of F^2^ > 2sigma(F^2^) is used only for calculating R-factors(gt) etc. and is not relevant to the choice of reflections for refinement. R-factors based on F^2^ are statistically about twice as large as those based on F, and R- factors based on ALL data will be even larger.

### Fractional atomic coordinates and isotropic or equivalent isotropic displacement parameters (Å^2^)

**Table:**

	*x*	*y*	*z*	*U*_iso_*/*U*_eq_	
I1	0.567756 (18)	0.313250 (10)	0.587900 (13)	0.03495 (7)	
S1	0.86223 (6)	0.46644 (4)	0.13656 (4)	0.02703 (12)	
F1	0.26520 (17)	0.54480 (10)	0.13600 (12)	0.0441 (4)	
C1	0.8607 (3)	0.52209 (14)	0.26168 (17)	0.0250 (4)	
O1	0.90716 (19)	0.63275 (10)	0.37911 (13)	0.0289 (3)	
O2	0.94638 (19)	0.38071 (11)	0.14941 (15)	0.0387 (4)	
C2	0.8029 (2)	0.49449 (14)	0.36768 (16)	0.0238 (4)	
C3	0.7257 (3)	0.42026 (14)	0.40952 (18)	0.0280 (4)	
H3	0.7021	0.3706	0.3650	0.034*	
C4	0.6852 (3)	0.42242 (15)	0.51872 (19)	0.0297 (5)	
C5	0.7189 (3)	0.49477 (16)	0.58547 (18)	0.0325 (5)	
H5	0.6886	0.4930	0.6601	0.039*	
C6	0.7957 (3)	0.56934 (15)	0.54554 (18)	0.0298 (5)	
C7	0.8348 (3)	0.56539 (14)	0.43646 (17)	0.0260 (4)	
C8	0.9211 (3)	0.60468 (14)	0.27344 (18)	0.0273 (4)	
C9	0.8290 (4)	0.64909 (17)	0.6148 (2)	0.0420 (6)	
H9A	0.9398	0.6685	0.6033	0.063*	
H9B	0.8138	0.6342	0.6919	0.063*	
H9C	0.7550	0.6966	0.5943	0.063*	
C10	0.9931 (3)	0.66853 (15)	0.1966 (2)	0.0378 (6)	
H10A	0.9995	0.6420	0.1238	0.057*	
H10B	1.1012	0.6842	0.2218	0.057*	
H10C	0.9264	0.7216	0.1933	0.057*	
C11	0.6519 (2)	0.44006 (14)	0.12914 (16)	0.0235 (4)	
C12	0.5369 (3)	0.50573 (15)	0.14214 (16)	0.0272 (4)	
H12	0.5655	0.5641	0.1624	0.033*	
C13	0.3799 (3)	0.48178 (16)	0.12415 (17)	0.0297 (5)	
C14	0.3321 (3)	0.39863 (17)	0.0960 (2)	0.0346 (5)	
H14	0.2218	0.3849	0.0854	0.042*	
C15	0.4494 (3)	0.33527 (16)	0.0835 (2)	0.0366 (5)	
H15	0.4197	0.2770	0.0638	0.044*	
C16	0.6107 (3)	0.35558 (16)	0.09950 (19)	0.0314 (5)	
H16	0.6910	0.3118	0.0901	0.038*	

### Atomic displacement parameters (Å^2^)

**Table:**

	*U*^11^	*U*^22^	*U*^33^	*U*^12^	*U*^13^	*U*^23^
I1	0.03228 (10)	0.03571 (10)	0.03690 (10)	0.00319 (6)	0.00514 (7)	0.01028 (6)
S1	0.0235 (3)	0.0315 (3)	0.0261 (3)	−0.0005 (2)	0.0032 (2)	−0.0058 (2)
F1	0.0332 (7)	0.0563 (9)	0.0430 (8)	0.0206 (7)	0.0027 (6)	−0.0041 (7)
C1	0.0253 (10)	0.0242 (10)	0.0254 (10)	0.0023 (8)	0.0001 (8)	−0.0024 (8)
O1	0.0346 (8)	0.0227 (7)	0.0293 (8)	0.0007 (6)	−0.0009 (7)	−0.0027 (6)
O2	0.0259 (8)	0.0370 (9)	0.0530 (11)	0.0077 (7)	−0.0050 (8)	−0.0157 (8)
C2	0.0239 (10)	0.0252 (10)	0.0224 (10)	0.0032 (8)	−0.0017 (8)	−0.0013 (8)
C3	0.0303 (11)	0.0250 (10)	0.0287 (11)	0.0007 (9)	0.0003 (9)	−0.0012 (8)
C4	0.0284 (11)	0.0300 (11)	0.0306 (11)	0.0036 (9)	0.0012 (9)	0.0065 (9)
C5	0.0383 (13)	0.0366 (12)	0.0227 (10)	0.0081 (10)	0.0016 (9)	0.0006 (9)
C6	0.0343 (12)	0.0301 (11)	0.0251 (10)	0.0081 (9)	−0.0045 (9)	−0.0034 (9)
C7	0.0280 (11)	0.0225 (10)	0.0275 (11)	0.0046 (8)	−0.0031 (9)	−0.0010 (8)
C8	0.0281 (10)	0.0252 (10)	0.0285 (11)	0.0029 (8)	−0.0001 (9)	−0.0018 (8)
C9	0.0573 (17)	0.0371 (13)	0.0316 (13)	0.0050 (12)	−0.0034 (12)	−0.0101 (11)
C10	0.0481 (15)	0.0257 (11)	0.0398 (14)	−0.0026 (10)	0.0077 (12)	0.0023 (10)
C11	0.0226 (10)	0.0282 (10)	0.0198 (9)	0.0014 (8)	0.0017 (8)	0.0016 (8)
C12	0.0313 (11)	0.0301 (11)	0.0202 (10)	0.0059 (9)	0.0028 (8)	−0.0014 (8)
C13	0.0260 (11)	0.0419 (13)	0.0214 (10)	0.0121 (9)	0.0035 (8)	0.0034 (9)
C14	0.0238 (11)	0.0448 (14)	0.0353 (12)	−0.0015 (10)	0.0010 (9)	0.0094 (10)
C15	0.0317 (12)	0.0314 (12)	0.0466 (14)	−0.0049 (10)	−0.0017 (11)	0.0078 (10)
C16	0.0264 (11)	0.0295 (12)	0.0383 (13)	0.0033 (9)	−0.0001 (9)	0.0042 (9)

### Geometric parameters (Å, º)

**Table:**

I1—C4	2.105 (2)	C6—C9	1.503 (3)
I1—O2^i^	3.2041 (16)	C8—C10	1.480 (3)
S1—O2	1.4862 (17)	C9—H9A	0.9800
S1—C1	1.750 (2)	C9—H9B	0.9800
S1—C11	1.798 (2)	C9—H9C	0.9800
F1—C13	1.360 (2)	C10—H10A	0.9800
C1—C8	1.358 (3)	C10—H10B	0.9800
C1—C2	1.449 (3)	C10—H10C	0.9800
O1—C8	1.368 (3)	C11—C16	1.376 (3)
O1—C7	1.381 (3)	C11—C12	1.392 (3)
C2—C7	1.392 (3)	C12—C13	1.374 (3)
C2—C3	1.396 (3)	C12—H12	0.9500
C3—C4	1.381 (3)	C13—C14	1.367 (3)
C3—H3	0.9500	C14—C15	1.380 (4)
C4—C5	1.397 (3)	C14—H14	0.9500
C5—C6	1.390 (3)	C15—C16	1.391 (3)
C5—H5	0.9500	C15—H15	0.9500
C6—C7	1.378 (3)	C16—H16	0.9500
			
C4—I1—O2^i^	165.03 (7)	C6—C9—H9B	109.5
O2—S1—C1	109.58 (10)	H9A—C9—H9B	109.5
O2—S1—C11	105.61 (10)	C6—C9—H9C	109.5
C1—S1—C11	98.11 (10)	H9A—C9—H9C	109.5
C8—C1—C2	107.24 (18)	H9B—C9—H9C	109.5
C8—C1—S1	122.30 (17)	C8—C10—H10A	109.5
C2—C1—S1	130.45 (16)	C8—C10—H10B	109.5
C8—O1—C7	106.85 (16)	H10A—C10—H10B	109.5
C7—C2—C3	119.29 (19)	C8—C10—H10C	109.5
C7—C2—C1	104.80 (18)	H10A—C10—H10C	109.5
C3—C2—C1	135.89 (19)	H10B—C10—H10C	109.5
C4—C3—C2	116.8 (2)	C16—C11—C12	121.8 (2)
C4—C3—H3	121.6	C16—C11—S1	117.57 (16)
C2—C3—H3	121.6	C12—C11—S1	120.35 (17)
C3—C4—C5	122.4 (2)	C13—C12—C11	116.6 (2)
C3—C4—I1	119.15 (17)	C13—C12—H12	121.7
C5—C4—I1	118.43 (17)	C11—C12—H12	121.7
C6—C5—C4	121.7 (2)	F1—C13—C14	118.2 (2)
C6—C5—H5	119.1	F1—C13—C12	117.7 (2)
C4—C5—H5	119.1	C14—C13—C12	124.1 (2)
C7—C6—C5	114.6 (2)	C13—C14—C15	117.8 (2)
C7—C6—C9	122.6 (2)	C13—C14—H14	121.1
C5—C6—C9	122.8 (2)	C15—C14—H14	121.1
C6—C7—O1	124.52 (19)	C14—C15—C16	120.9 (2)
C6—C7—C2	125.1 (2)	C14—C15—H15	119.5
O1—C7—C2	110.33 (18)	C16—C15—H15	119.5
C1—C8—O1	110.78 (19)	C11—C16—C15	118.9 (2)
C1—C8—C10	133.5 (2)	C11—C16—H16	120.5
O1—C8—C10	115.72 (19)	C15—C16—H16	120.5
C6—C9—H9A	109.5		
			
O2—S1—C1—C8	123.85 (19)	C3—C2—C7—C6	−0.2 (3)
C11—S1—C1—C8	−126.32 (19)	C1—C2—C7—C6	178.1 (2)
O2—S1—C1—C2	−55.4 (2)	C3—C2—C7—O1	−178.33 (18)
C11—S1—C1—C2	54.4 (2)	C1—C2—C7—O1	0.0 (2)
C8—C1—C2—C7	0.0 (2)	C2—C1—C8—O1	0.1 (2)
S1—C1—C2—C7	179.29 (17)	S1—C1—C8—O1	−179.29 (15)
C8—C1—C2—C3	177.8 (2)	C2—C1—C8—C10	−178.4 (2)
S1—C1—C2—C3	−2.8 (4)	S1—C1—C8—C10	2.2 (4)
C7—C2—C3—C4	0.1 (3)	C7—O1—C8—C1	−0.1 (2)
C1—C2—C3—C4	−177.6 (2)	C7—O1—C8—C10	178.6 (2)
C2—C3—C4—C5	0.0 (3)	O2—S1—C11—C16	−21.3 (2)
C2—C3—C4—I1	179.77 (15)	C1—S1—C11—C16	−134.30 (18)
O2^i^—I1—C4—C3	34.5 (4)	O2—S1—C11—C12	165.15 (17)
O2^i^—I1—C4—C5	−145.8 (2)	C1—S1—C11—C12	52.11 (19)
C3—C4—C5—C6	0.0 (4)	C16—C11—C12—C13	0.2 (3)
I1—C4—C5—C6	−179.77 (17)	S1—C11—C12—C13	173.49 (15)
C4—C5—C6—C7	−0.1 (3)	C11—C12—C13—F1	−179.81 (18)
C4—C5—C6—C9	178.2 (2)	C11—C12—C13—C14	0.7 (3)
C5—C6—C7—O1	178.1 (2)	F1—C13—C14—C15	179.6 (2)
C9—C6—C7—O1	−0.2 (3)	C12—C13—C14—C15	−0.9 (4)
C5—C6—C7—C2	0.1 (3)	C13—C14—C15—C16	0.2 (4)
C9—C6—C7—C2	−178.1 (2)	C12—C11—C16—C15	−0.8 (3)
C8—O1—C7—C6	−178.1 (2)	S1—C11—C16—C15	−174.31 (19)
C8—O1—C7—C2	0.1 (2)	C14—C15—C16—C11	0.6 (4)

Symmetry code: (i) *x*−1/2, −*y*+1/2, *z*+1/2.

### Hydrogen-bond geometry (Å, º)

**Table:**

*D*—H···*A*	*D*—H	H···*A*	*D*···*A*	*D*—H···*A*
C14—H14···O2^ii^	0.95	2.43	3.292 (3)	151

Symmetry code: (ii) *x*−1, *y*, *z*.
